# Left ventricular apical hypertrophy in a transplanted heart: a case report

**DOI:** 10.1186/s12872-019-1069-4

**Published:** 2019-04-03

**Authors:** Uzochukwu Ibe, Kathir Balakumaran, Sabeena Arora

**Affiliations:** 1 Department of Internal Medicine, St. Mary’s hospital, 56 Franklin street, Waterbury, CT 06716 USA; 2 Department of Cardiology, University of Connecticut, 263 Farmington avenue, Farmington, CT 06030 USA; 3 Department of Cardiology, St Francis Hospital and Medical Center, 114 Woodland St, Hartford, CT 06105 USA

**Keywords:** Apical hypertrophic cardiomyopathy, Transplanted heart, Left ventricular apical hypertrophy, Transplant

## Abstract

**Background:**

Left ventricular apical hypertrophic cardiomyopathy is a rare presentation of hypertrophic cardiomyopathy associated with thickening of the apical segment of the left ventricle. It was initially described in Japan in 1976 and is characterized by electrocardiogram findings showing giant T wave inversions in the precordial leads as well as a spade shaped appearance of the apical cavity on imaging (Abugroun et al., Cardiol Res 8:265-268, 2017).

In this case, we present a patient with a heart transplant with a stable post-transplant course who was found to have apical hypertrophic cardiomyopathy. There have been a few cases of apical hypertrophy in a transplanted heart documented in the literature. Making this case even more unique is that this presentation is evident 17 years after heart transplantation.

**Case presentation:**

Fifty-four year-old male with a history of orthotropic heart transplant in 2001 on immunosuppressive therapy presented with palpitations and associated lightheadedness. He had a blood pressure of 184/89 mmHg on arrival but otherwise had stable vital signs and physical examination. Cardiac biomarkers revealed a CK of 59 U/L and a troponin of 0.11NG/ML(normal < 0.04NG/ML). B type natriuretic peptide was 371 PG/ML(normal 0-100PG/ML). Routine laboratory studies demonstrated normal sodium, magnesium, serum creatinine, and a potassium of 3.3 mmol/L(normal 3.5–5.1 mmol/L). His hemoglobin and hematocrit were normal. His EKG showed sinus rhythm with old T wave inversions in the anterior and lateral leads. Echocardiogram revealed a left ventricular ejection fraction of 55–65%, left posterior wall of 1.3 cm and interventricular septal wall 1.2 cm, thickened trabeculated apex, with severely dilated left atrium. He had a stress test that showed mild inferior wall thinning and a cardiac MRI performed to further evaluate apical hypertrophy revealed prominent apical hypertrophy of the left ventricle with near obliteration of the apical cavity. He had no events on cardiac monitoring and was discharged with close followup with the transplant team.

**Conclusion:**

While there are many etiologies of ApHCM, it has not been well described in transplanted patients who are on chronic immunosuppressive therapy. It is unclear if these groups of patients are at an increased risk of developing this condition. The literature suggests that ApHCM is associated with a being prognosis but there is new data suggesting increased mortality in a subset of patients with this condition.

## Background

Left ventricular apical hypertrophic cardiomyopathy is a rare presentation of hypertrophic cardiomyopathy associated with thickening of the apical segment of the left ventricle. It was initially described in Japan in 1976 [1] and is characterized by electrocardiogram findings showing giant T wave inversions in the precordial leads as well as a spade shaped appearance of the apical cavity on imaging [2]. In western countries, the reported incidence is between 1 and 2% [3].

In this case, we present a patient with a heart transplant with a stable post-transplant course who was found to have apical hypertrophic cardiomyopathy. There have been a few cases of apical hypertrophy in a transplanted heart documented in the literature. Making this case even more unique is that this presentation is evident 17 years after transplantation.

## Case presentation

A 54 year-old male with a history of orthotropic heart transplant in 2001 for non-ischemic cardiomyopathy presented to the hospital after experiencing intermittent palpitations with associated lightheadedness of one hour duration. He denied any chest pain or shortness of breath, orthopnea or paroxysmal nocturnal dyspnea. At baseline, he described activities consistent with New York Heart Association Class 2. He had recently completed a 4 day course of Prednisone for a gout flare-up. Additional medical history was notable for benign prostatic hyperplasia and hyperlipidemia. Medications included Atorvastatin, Cyclosporine, Finasteride, Levothyroxine, Mirtazapine, Mycophenolate, Omeprazole, Prednisone, Quetiapine, Ranitidine, Tamsulosin, Zolpidem. He had allergies to Azithromycin. He denied tobacco or illicit drug use and alcohol consumption.

**Fig. 1:**
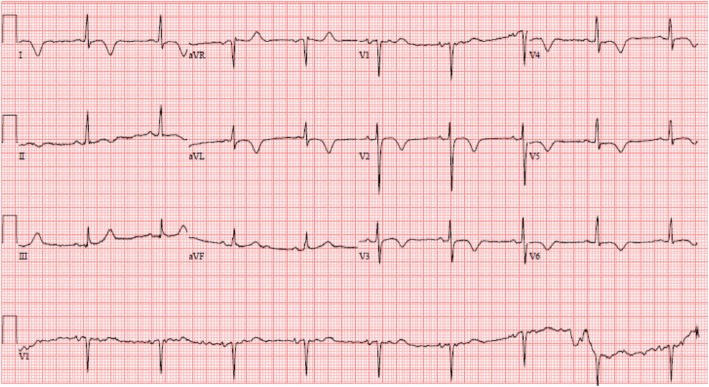
Electrocardiogram revealing sinus rhythm with normal axis, and diffuse T wave inversions in leads V2 to V6

**Fig. 2:**
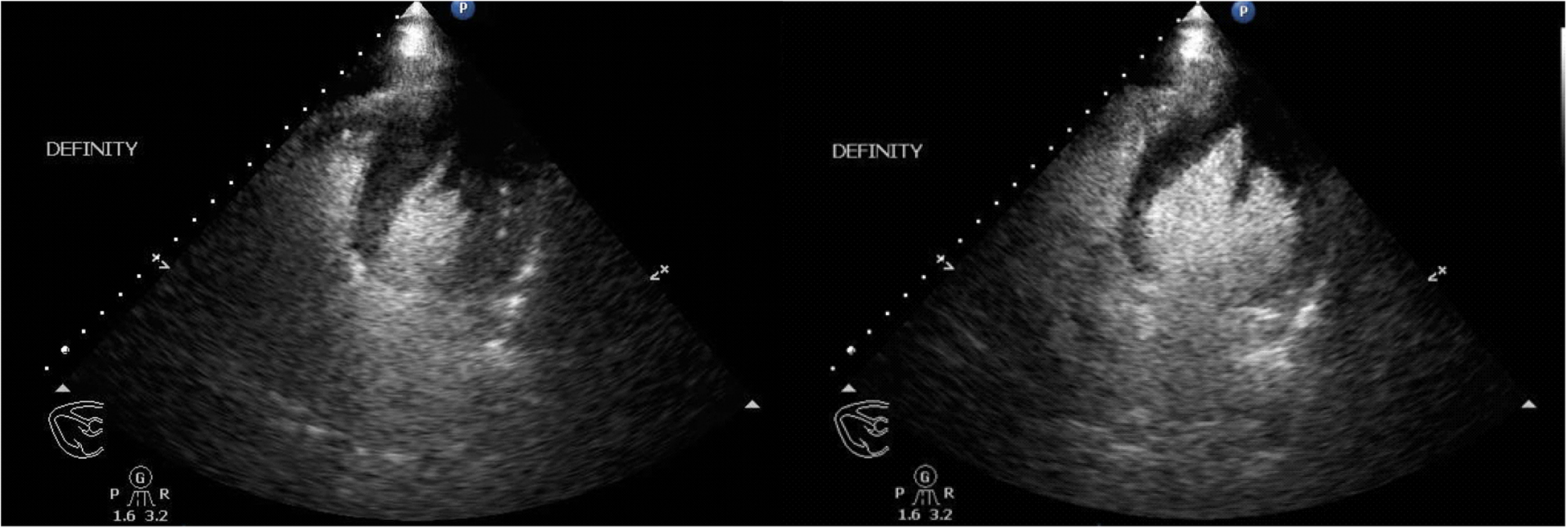
Left Ventricular Apical Hypertrophy noted on transthoracic echocardiogram in 4 chamber apical view with Definity contrast

**Fig. 3:**
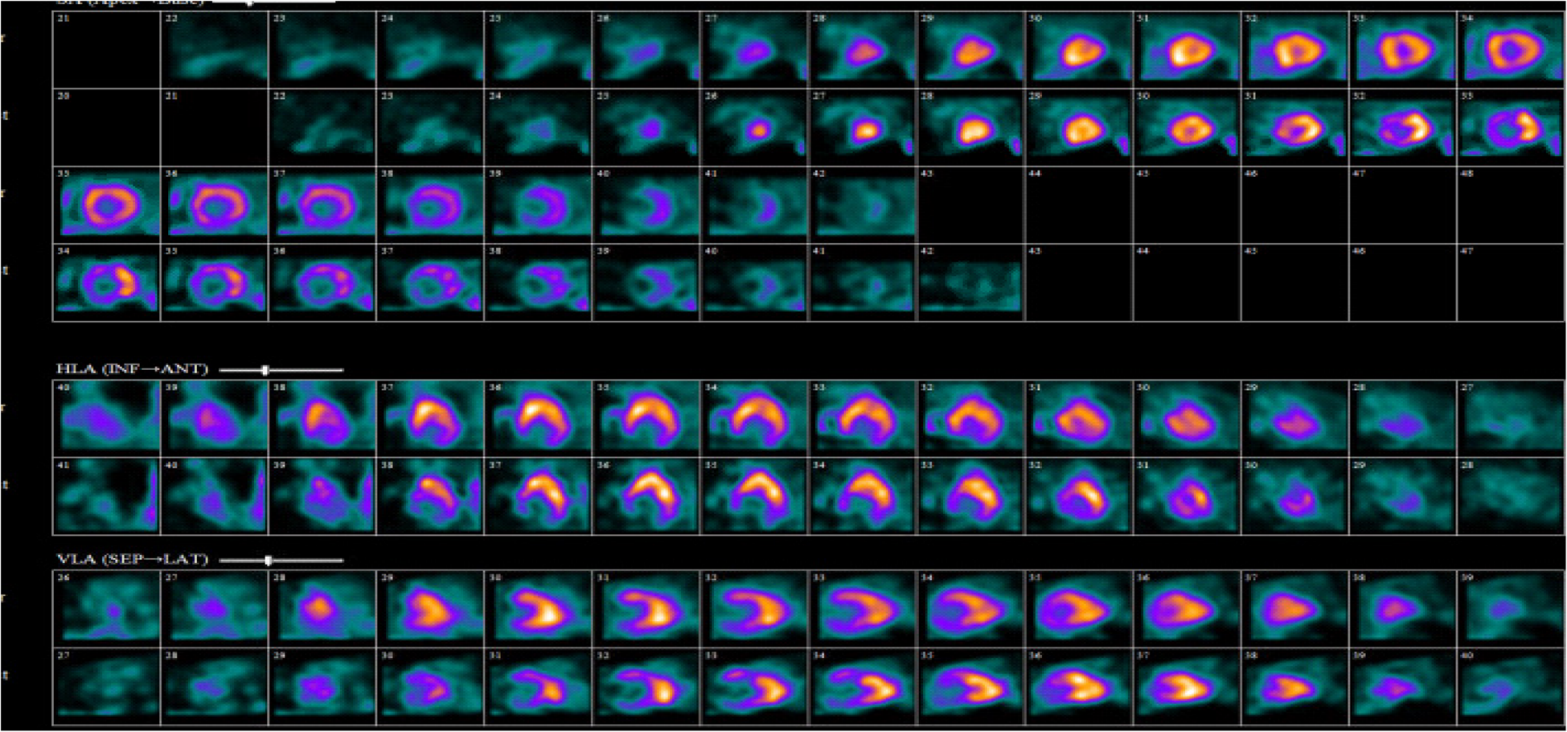
Myocardial perfusion imaging in comparing rest (top row) and stress (bottom row) revealing no significant reversible ischemia

**Fig. 4:**
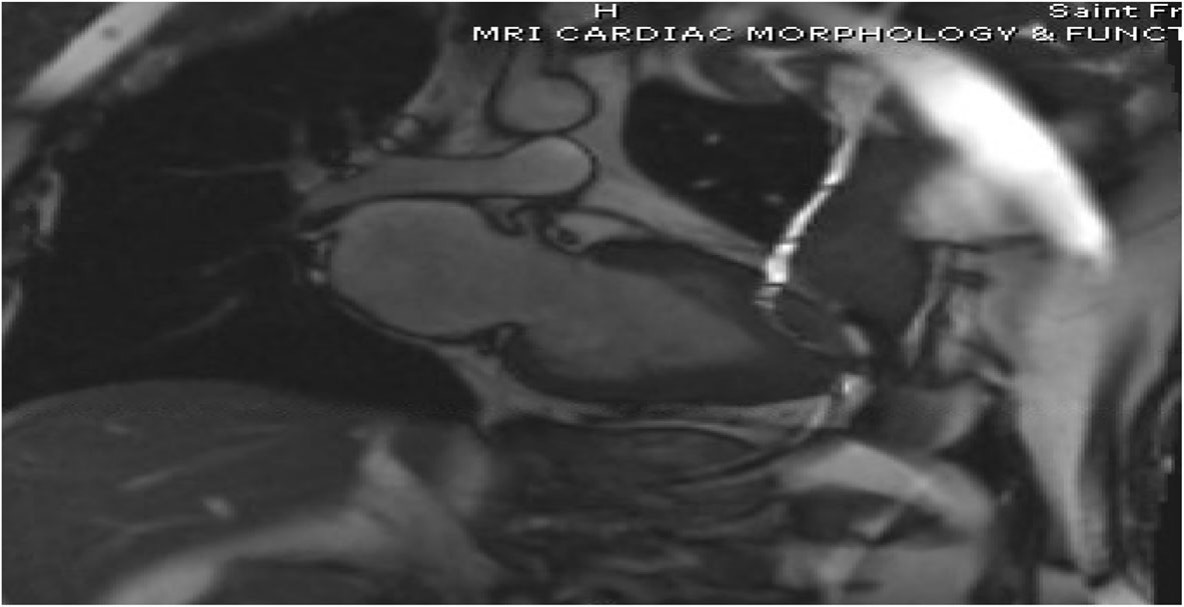
MRI imaging revealing severely thickened apical myocardium with “spade” appearance

On arrival, he was afebrile, heart rate was 54 bpm, blood pressure was 184/89 mmHg, and oxygen saturation was 98% on ambient air. His physical examination was unremarkable and he appeared clinically euvolemic. Cardiac biomarkers revealed a CK of 59 U/L and a troponin of 0.11NG/ML(normal < 0.04NG/ML). B type natriuretic peptide was 371 PG/ML(normal 0-100PG/ML). Routine laboratory studies demonstrated normal sodium, magnesium, serum creatinine, and a potassium of 3.3 mmol/L(normal 3.5–5.1 mmol/L). His hemoglobin and hematocrit were normal. An electrocardiogram revealed a normal sinus rhythm with T wave inversions in the anterior and lateral leads which were unchanged compared to prior ECG (Fig. 1). He had no evidence of arrhythmias on cardiac monitoring. Transthoracic echocardiogram revealed left ventricular ejection fraction of 55–65%, left posterior wall of 1.3 cm and interventricular septal wall 1.2 cm, thickened trabeculated apex, with severely dilated left atrium and mildly hypertrophied right ventricle (Fig. 2). He underwent a nuclear stress test that revealed mild inferior wall thinning and normal wall motion (Fig. 3). A cardiac MRI was performed to further evaluate apical hypertrophy based on his echocardiogram findings. This revealed prominent apical hypertrophy of the left ventricle with near obliteration of the apical cavity (Fig. 4). His cardiac biomarkers trended down to normal and he had no evidence of arrhythmias on continuous cardiac monitoring so he was discharged from the hospital with close follow up with his heart transplant team.

## Discussion and conclusions

Apical Hypertrophic Cardiomyopathy (ApHCM) is a relatively rare variant of hypertrophic cardiomyopathy associated with thickening of the apical segment of the left ventricle leading to a ‘spade-shaped’ small cavity. Initially described in Japan, it makes up approximately 15–25% of all hypertrophic cardiomyopathies and is more commonly seen in men [3].

The hallmarks include giant T wave inversions in the precordial leads on electrocardiogram and the aforementioned spade like appearance of the left ventricular cavity [1, 2]. Most of the available data based on studies done in the Japanese population have suggested a benign prognosis in patients with ApHCM. Clinically, the manifestations can vary from apical aneurysms, atrial tachycardia or atrial fibrillation, ventricular arrhythmias and sudden cardiac death. About 54% of patients are asymptomatic [4]. Nevertheless, one third of patients experience serious cardiovascular complications, such as myocardial infarction and arrhythmias [5].

In a transplanted heart, various causes of hypertrophy exist. These include long standing hypertension and pressure overload, medications like tacrolimus, metabolic disorders and donor hypertrophic cardiomyopathy [6].

This patient’s case is rather peculiar because he had a heart transplant seventeen years ago and has been maintained on immunosuppressive therapy with a rather benign post-transplant course. He had an echocardiogram dating back five years ago that had no evidence of ApHCM so it is safe to assume that the pre-transplant donor echo was unremarkable. It is unclear if there was any history of HCM or sudden cardiac death in donor’s family. He had well controlled blood pressures and had no evidence of ischemia or valvular disease on non-invasive testing. It is certainly possible that this is an idiopathic finding.

The finding of apical hypertrophic cardiomyopathy in this patient likely does not suggest an increase in mortality or morbidity due to its historically benign course. However, recent studies have shown increased morbidity and mortality in this subset of patients [4]. Factors like advanced age, hypertension, diabetes, baseline atrial fibrillation, left atrial volume index and apical aneurysm on cardiac magnetic resonance are predictors of poor prognosis. A retrospective study of 105 patients done in Toronto from 1975 to 2000 showed that in North American patients, ApHCM was not associated with an increased risk of sudden cardiac death but one third of patients experienced myocardial infarctions and arrhythmias [6]. Probability of survival without morbid events was 74% at 15 years [6]. Since our patient was rather asymptomatic up to this point, he was discharged on his preexisting medications with outpatient follow-up.

In conclusion, apical hypertrophic cardiomyopathy is a well described benign variant of hypertrophic cardiomyopathy. However, it has not been well described in a patient with previous heart transplant. The etiology of ApHCM in this patient is thought to be idiopathic and less likely related to his long term immunosuppressive therapy. It is unclear if the prognosis differs in this group of patients but studies done to assess long term outcome have shown a benign prognosis in terms of cardiovascular mortality.

## Abbreviations

ApHCM: Apical hypertrophic cardiomyopathy
